# Replacement of Dietary Fishmeal Protein with Degossypolized Cottonseed Protein on Growth Performance, Nonspecific Immune Response, Antioxidant Capacity, and Target of Rapamycin Pathway of Juvenile Large Yellow Croaker (*Larimichthys crocea*)

**DOI:** 10.1155/2022/8529556

**Published:** 2022-09-22

**Authors:** Sheng Chen, Yuhang Tang, Zhou Zhang, Jichang Zheng, Yuliang He, Zhen Wang, Kangsen Mai, Qinghui Ai

**Affiliations:** ^1^Key Laboratory of Aquaculture Nutrition and Feed, Ministry of Agriculture and Rural Affairs, The Key Laboratory of Mariculture, Ministry of Education, Ocean University of China, 5 Yushan Road, Qingdao, Shandong 266003, China; ^2^Laboratory for Marine Fisheries Science and Food Production Processes, Qingdao National Laboratory for Marine Science and Technology, 1 Wenhai Road, Qingdao, Shandong 266237, China

## Abstract

A 70-day feeding experiment was carried out to assess the replacement of dietary fishmeal (FM) protein with degossypolized cottonseed protein (DCP) on large yellow croaker (*Larimichthys crocea*) with initial body weight (13.09 ± 0.50 g). Five isonitrogenous and isolipidic diets replaced fishmeal protein with 0%, 20%, 40%, 60%, and 80% DCP were formulated and named as FM (the control group), DCP20, DCP40, DCP60, and DCP80, respectively. Results displayed that weight gain rate (WGR) and specific growth rate (SGR) in the DCP20 group (263.91% and 1.85% d^−1^) were significantly increased compared with the control group (194.79% and 1.54% d^−1^) (*P* < 0.05). Furthermore, fish fed the diet with 20% DCP significantly increased the activity of hepatic superoxide dismutase (SOD) compared with the control group (*P* < 0.05). Meanwhile, the content of hepatic malondialdehyde (MDA) in the DCP20, DCP40, and DCP80 groups was significantly lower than that in the control group (*P* < 0.05). The activity of intestinal trypsin in the DCP20 group was significantly degraded compared with that in the control group (*P* < 0.05). The transcription of hepatic proinflammatory cytokine genes (interleukin-6 (*il-6*); tumor necrosis factor-*α* (*tnf-α*); and interferon-*γ* (*ifn-γ*)) in the DCP20 and DCP40 groups was significantly upregulated compared with that in the control group (*P* < 0.05). As to the target of rapamycin (TOR) pathway, the transcription of hepatic target of rapamycin (*tor*) and ribosomal protein (*s6*) was significantly up-regulated, while the transcription of hepatic eukaryotic translation initiation factor 4E binding protein 1 (*4e-bp1*) gene was significantly downregulated in the DCP group compared with the control group (*P* < 0.05). In summary, based on the broken line regression model analysis of WGR and SGR against dietary DCP replacement levels, the optimal replacement level was recommended to be 8.12% and 9.37% for large yellow croaker, respectively. These results revealed that FM protein replaced with 20% DCP could promote digestive enzyme activities and antioxidant capacity and further activate immune response and the TOR pathway so that growth performance of juvenile large yellow croaker was improved.

## 1. Introduction

Fishmeal (FM) protein, characterized by easy availability, balanced amino acid profile, and high digestibility, is regarded as the main protein source in aquafeed [1–3]. The rapid development of aquaculture well boosts the feed industry and sharply increases the demand for FM. However, FM production is decreasing year by year. This maladjustment between provision and requirement leads to the high price of FM, which urges people to explore suitable substitutes for protein sources. Plant-derived materials are considered the first choice of high-quality protein sources to replace FM due to high yield, easy availability, and low price. However, they are limited as feed ingredients due to imperfect amino acid compositions and the existence of antinutritional factors (ANFs) [4, 5]. Free gossypol is the main ANF in cottonseed by-products. Degossypolized cottonseed protein (DCP), which was obtained by one-step low-temperature leaching and two-solvent stepwise extraction process to remove the majority of free gossypol, is a high-quality feed protein source with low free gossypol and relatively complete amino acid profile. Thus, DCP has been used to substitute FM protein in several fishes [6, 7].

Previous studies have shown that FM protein replaced with DCP will have effects on antioxidant activity, digestive enzyme activity, and immune response in some aquatic animals. Zhao et al. [8] indicated that FM protein replaced with concentrated dephenolized cottonseed protein (CDCP) enhanced the antioxidant activity of rain trout (*Oncorhynchus mykiss*). The study in South American white shrimp (*Litopenaeus vannamei*) found that intestinal digestive enzyme activities were significantly improved due to replacement of FM protein with cottonseed protein concentrate (CPC) [9]. In addition, it was indicated that high content of low-gossypol cottonseed protein concentrate (CPC) induced inflammation in swimming crab (*Portunus trituberculatus*) [10].

The target of rapamycin (TOR) pathway was involved as a crucial regulator in several extracellular and intracellular signaling pathways, such as growth factors and nutritional status in mammals [11]. Protein synthesis is closely relevant to organismal growth and homeostasis, which can be regulated by the TOR pathway [12]. The connection between activation of the TOR pathway and promotion of growth performance had been revealed in blunt snout bream (*Megalobrama amblycephala*) [13], gibel carp (*Carassius auratus gibelio* var. CAS V) [14], and jian carp (*Cyprinus carpio* var. Jian) [15], which indicated that the TOR pathway could profoundly govern the growth of fish similar to mammals. Some studies on large yellow croaker larvae suggested that dietary size-fractionated fish hydrolysates and amino acids could not significantly impact the TOR pathway [16, 17]. However, other studies of different aquatic animals indicated that the transcription of the TOR pathway-related genes was affected by supplementing some amino acids to diets [14, 18, 19]. These different results could be attributed to the differences in trial animals of age and tissue. So far, little data is available on whether the TOR pathway is regulated by DCP in juvenile large yellow croaker and needs further investigation.

Large yellow croaker (*Larimichthys crocea*) is a crucial marine fish with the highest yield and great economic value in China [20, 21], and its output has approached 254,062 tons in 2020 [22]. With the continuous expansion of the cultured scale, the demand for the production capacity of FM in feed is also gradually increasing. However, few studies on large yellow croaker focused on effects of FM protein replaced with dietary DCP. Thus, the feasibility of FM protein replaced with dietary DCP on growth performance, antioxidant capacity, digestive enzyme activities, the TOR pathway, and immune response of juvenile was assessed in the present study, which could fill the gap in the research of replacing dietary FM protein with DCP of large yellow croaker and provide a reference for its application in aquaculture.

## 2. Materials and Methods

### 2.1. Experimental Diets

Five isonitrogenous (41% crude protein content) and isolipidic (12% crude lipid content) diets replacing 0% (the control group), 20%, 40%, 60%, and 80% FM protein with DCP were formulated (Table 1). Based on the control diet, graded levels of DCP (89.00, 175, 264, and 349 g/kg dry matter) were, respectively, added to formulate experimental diets. The detailed production process of feed pellets was followed on the previous research of Li et al. [23]. Then, feed pellets were packed in plastic sealed bags and stored at -20°C until use.

### 2.2. Experimental Procedures

All experimental juvenile large yellow croakers were fed the control diets for two weeks to acclimatize the trial conditions. Nine hundred fish with mean body weight (13.09 ± 0.50 g) were randomly put in 15 seawater cages (1.0 m × 1.0 m × 1.5 m) after being starved for 24 h. Each dietary treatment was set three biological repeats. During the feeding trial, fish were fed once in the morning (06:00) and evening (17:00) for 10 weeks. The management of water quality parameters was measured in a general range (temperature: 19.8-22.5°C, salinity: 25.7-29.7‰, and oxygen level: 6.0-7.2 mg/L).

### 2.3. Sample Collection

The experimental fish were treated by eugenol (1 : 10,000) narcosis to collect samples, which were fasted for 24 h. The amount and weight of survival fish from each experimental seawater cage were, respectively, recorded and weighted. Three fish randomly from each seawater cage were used for whole body composition analysis. Samples of the liver, intestine, and serum were collected from eight randomly fish in each seawater cage, and then, the samples of liver and intestine were packed into 10 mL tubes, immediately stored in liquid nitrogen for further analysis. Meanwhile, the sample of serum was injected into a 1.5 mL centrifuge tube and stored in liquid nitrogen for further analysis. Subsequently, the morphological parameters were calculated based on the recorded data of six fish randomly selected from each seawater cage.

### 2.4. Body Composition Analysis

The approximate chemical composition of whole body in trial fish samples was analyzed following by the method of AOAC [24].

### 2.5. Serum Biochemical Analysis

The contents of serum triglyceride (TG), total cholesterol (TC), activities of serum alanine transaminase (ALT), aspartate transaminase (AST), and alkaline phosphatase (ALP) were tested by kits from Nanjing Jiancheng Biological Engineering Institute Co., Ltd. The detailed experimental steps refer to the instruction attached to the kit.

### 2.6. Hepatic Antioxidant Capacity Analyses

Activities of hepatic superoxide dismutase (SOD) and catalase (CAT), total antioxidant capacity (T-AOC), and the content of hepatic malondialdehyde (MDA) were detected by kits from Nanjing Jiancheng Biological Engineering Institute Co., Ltd. The detailed experimental steps were presented in the instruction attached to the kit.

### 2.7. Intestinal Digestive Enzyme Activities

Activities of intestinal lipase (LPS), amylase (AMS), and trypsin were quantified by kits from Nanjing Jiancheng Biological Engineering Institute Co., Ltd. The specific experimental operations were followed by the instruction attached to the kit.

### 2.8. RNA Extraction, cDNA Synthesis, and Real-Time Quantitative PCR (RT-qPCR)

Samples of experimental fish liver were cut and added Trizol Reagent (Takara) in order to extract total RNA. Then, RNA was reversed transcribe into cDNA using Prime Script-RT reagent Kit (Takara, Japan). The amplification system and program of RT-qPCR were set up referring to the published study in our lab [25]. The primer sequences (Table 2) of *il-1β*, *il-6*, *il-8*, *il-10*, *tnf-α*, *inf-γ*, *tor*, *s6*, *4e-bp1*, and *β-actin* for RT-qPCR were designed by Primer Premier 5.0 software. The amplification efficiency and product specificity were verified followed by the operation of Li et al. [21]. The relative expression of genes was calculated by the 2^-*ΔΔ*Ct^ method.

### 2.9. Calculations and Statistical Analysis

The following are the calculations and statistical analysis:

Survival rate (SR, %) = the initial amount of experimental fish/the final amount of survival fish × 100.

Weight gain rate (WGR, %) = (finial wet weight of fish–initial wet weight of fish)/initial wet weight of fish × 100.

Specific growth rate (SGR, %day^−1^) = [Ln (finial wet weight of fish) − Ln (initial wet weight of fish)] × 100/trial days.

Viscerosomatic index (VSI, %) = *W*_*v*_/finial wet weight of fish × 100.

Hepatosomatic index (HSI, %) = *W*_*l*_/finial wet weight of fish × 100.

In the above, *W*_*v*_ and *W*_*l*_ were visceral weight and liver weight (g/wet weight) of fish, respectively.

SPSS 25.0 software (IBM, USA) was used to analyze all experimental data. The method of one-way analysis of variance (ANOVA) was used to analysis all data, and then, Tukey's test was used to determine the differences of all data between dietary treatments. All data of the present experimental results were presented as the means ± SEM (standard error of the mean). *P* < 0.05 was considered significant.

## 3. Results

### 3.1. Survival Rate, Growth Performance, and Body Indexes

**Figure 1:**
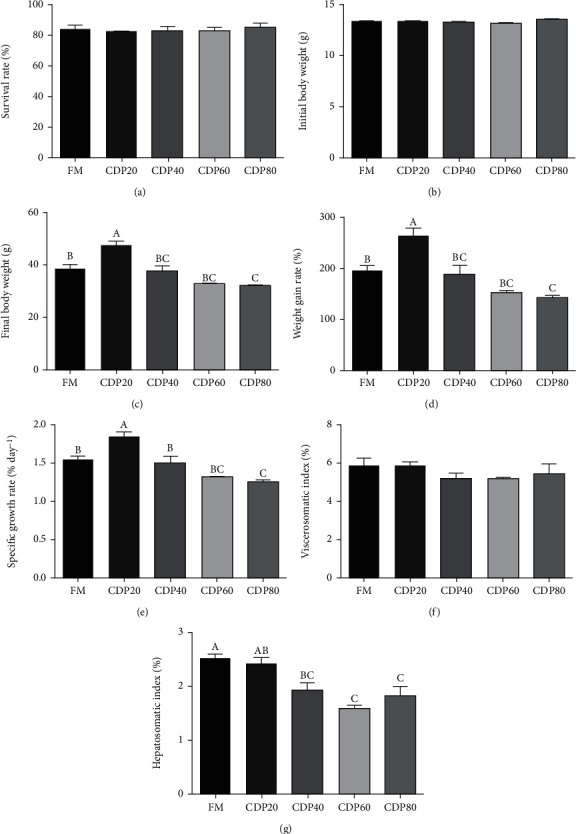
Effect of degossypolized cottonseed protein on survival rate, growth performance, and body indexes of juvenile fed the trial diets (means ± SEM, *n* = 3). No repetition of letters in the equal index means significant differences (*P* < 0.05).

**Figure 2:**
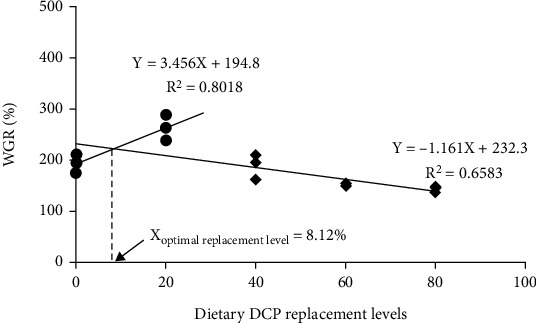
The broken line model for the relationship between WGR and dietary DCP replacement levels of juvenile large yellow croaker fed the trial diets.

**Figure 3:**
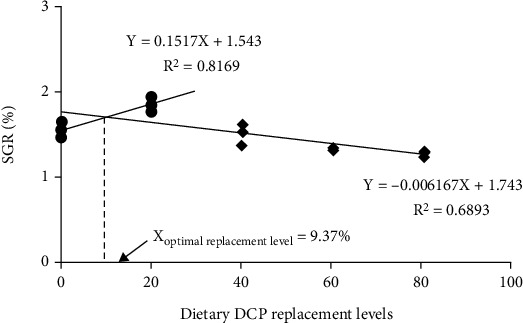
The broken line model for the relationship between SGR and dietary DCP replacement levels of juvenile large yellow croaker fed the trial diets.

As dietary DCP replacement levels increased from 0 to 20%, the specific growth rate (SGR) significantly elevated and then significantly decreased from 1.85 to 1.27% d^−1^ with increasing dietary DCP levels (Figure 1(e)). Consistent with the results of SGR, the weight gain rate (WGR) reached the highest level in the DCP20 group and then degraded with increasing dietary CDP replacement levels (*P*<0.05) (Figure 1(d)). Based on the broken line regression model of WGR and dietary DCP replacement levels, it was indicated that the optimal replacement level was 8.12% (Figure 2), while the analysis of the above model between SGR and dietary DCP replacement levels showed that the optimal replacement level was 9.37% (Figure 3). However, dietary DCP replacement levels did not significantly impact the survival rate (SR) (*P* > 0.05) (Figure 1(a)). In terms of body indexes, a decreasing trend was observed in HSI (from 2.49 to 2.41%) and then significantly degraded with increasing dietary DCP levels (*P* < 0.05) (Figure 1(g)). Nevertheless, dietary DCP replacement levels did not significantly impact VSI in the present study (*P* > 0.05) (Figure 1(f)).

### 3.2. Body Composition

As dietary DCP replacement levels increased, the whole fish body content of moisture, crude protein, and crude lipid was not significantly affected (*P* > 0.05) (Table 3).

### 3.3. Serum Biochemical Indexes

Dietary DCP replacement levels did not significantly impact the contents of serum triglyceride (TG) and total cholesterol (TC) contents in the present study (*P* > 0.05). As dietary DCP replacement levels increased from 20% to 60%, the activity of serum alanine transaminase (ALT) in fish was significantly elevated compared with the control group (*P* < 0.05). Simultaneously, the activity of aspartate transaminase (AST) in the DCP20 group was significantly elevated compared with that in the control group (*P* < 0.05). Moreover, as dietary DCP replacement levels increased, the activity of serum alkaline phosphatase (ALP) in fish appeared to have no significant difference (*P* > 0.05) (Table 4).

### 3.4. Hepatic Antioxidant Capacity

**Figure 4:**
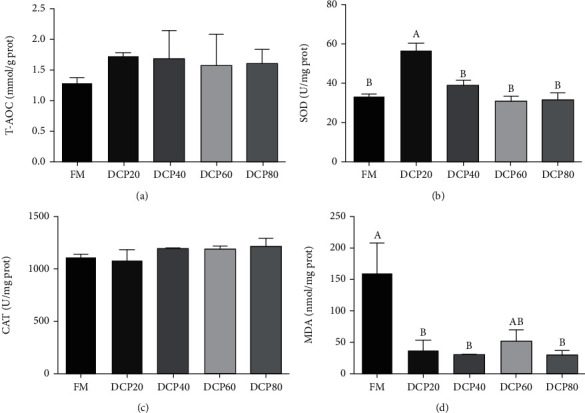
Effect of degossypolized cottonseed protein on hepatic antioxidant capacity of juvenile fed the trial diets (means ± SEM, *n* = 3). T-AOC: total antioxidant capacity; SOD: superoxide dismutase; CAT: catalase; MDA: malondialdehyde. No repetition of letters in the equal index means significant differences (*P* < 0.05).

As dietary DCP replacement levels elevated, hepatic antioxidant capacity of fish was degraded. The activity of superoxide dismutase (SOD) in the DCP20 group was significantly elevated compared with that in the control group and then significantly degraded as increasing dietary DCP replacement levels (*P* < 0.05) (Figure 4(b)). Meanwhile, the concentration of malondialdehyde (MDA) in the DCP20, DCP40, and DCP80 groups was significantly lower than that in the control group (*P* < 0.05) (Figure 4(d)). However, total antioxidant capacity (T-AOC) and activity of catalase (CAT) were not significantly affected by dietary DCP replacement levels (*P* > 0.05) (Figures 4(a) and 4(c)).

### 3.5. Intestinal Digestive Enzyme Activities

**Figure 5:**
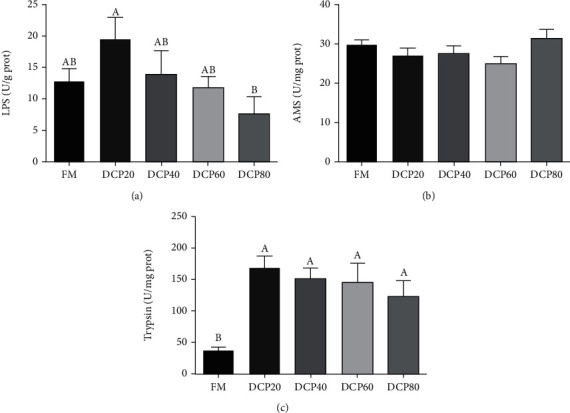
Effect of degossypolized cottonseed protein on intestinal digestive enzyme activities of juvenile fed the trial diets (means ± SEM, *n* = 3). LPS: lipase; AMS: amylase. No repetition of letters in the equal index means significant differences (*P* < 0.05).

In the present study, a decreasing trend was observed in the activity of intestinal lipase (LPS) in fish fed diets with from 20% to 80% DCP, but dietary DCP replacement levels did not significantly impact the trend of change (*P* > 0.05) (Figure 5(a)). Meanwhile, as dietary DCP levels increased, the activity of intestinal trypsin in the DCP20 group was significantly elevated compared with the control group (*P* < 0.05) (Figure 5(c)). However, dietary DCP replacement levels did not significantly affect the activity of amylase (AMS) compared with the control group (*P* > 0.05) (Figure 5(b)).

### 3.6. Relative Gene Expression Related to Inflammation in the Liver

**Figure 6:**
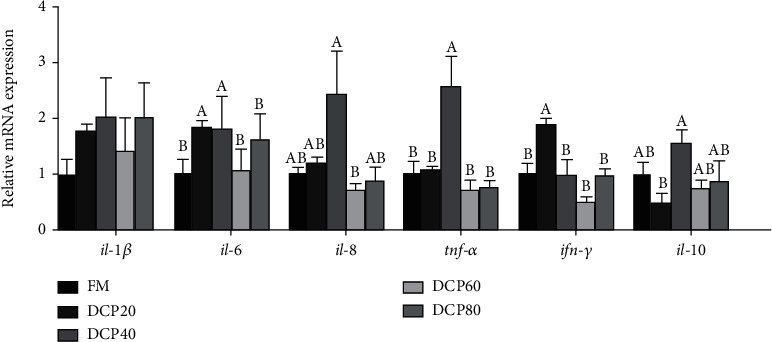
Relative expression of genes related to hepatic inflammatory cytokines of juvenile fed the trial diets (means ± SEM, *n* = 3). *il-1β*: interleukin-1*β*; *il-6*: interleukin-6; *il-8*: interleukin-8; *il-10*: interleukin-10; *tnf-α*: tumor necrosis factor-*α*; *ifn-γ*: interferon-*γ*. No repetition of letters in the equal index means significant differences (*P* < 0.05).

The transcription of proinflammatory cytokines was significantly increased in the DCP20 (*il-6* and *ifn-γ*) and DCP40 (*il-6* and *tnf-α*) groups compared with the control group (*P* < 0.05), but the transcription of *il-6*, *ifn-γ*, and *tnf-α* in the DCP60 and DCP80 groups was not significantly impacted compared with the control group (*P* > 0.05). Meanwhile, compared with the control group, the transcription of anti-inflammatory (*il-10*) in the DCP20 group was observed a degraded trend (*P* > 0.05) (Figure 6).

### 3.7. Relative Gene Expression Related to Protein Metabolism in the Liver

**Figure 7:**
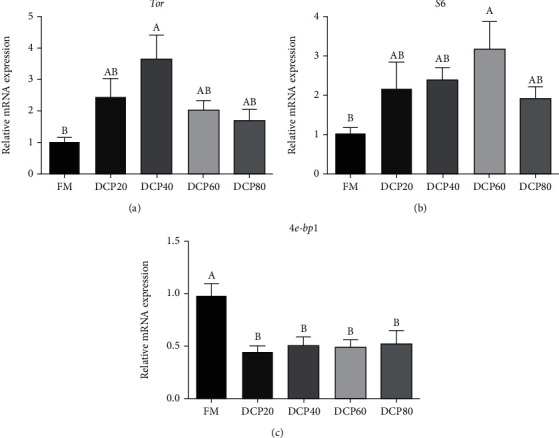
Relative expression of genes related to the TOR pathway of juvenile fed the trial diets (means ± SEM, *n* = 3). *tor*: target of rapamycin; *s6*: ribosomal protein S6; *4e-bp1*: eukaryotic initiation factor4E binding protein 1. No repetition of letters in the equal index means significant differences (*P* < 0.05).

The transcription of *tor* was significantly elevated in the DCP40 group (*P* < 0.05), while the transcription of *tor* in the DCP20, DCP60, and DCP80 groups was not significantly impacted compared with the control group (*P* > 0.05) (Figure 7(a)). Meanwhile, the transcription of *s6* was significantly increased in the DCP60 group (*P* < 0.05) (Figure 7(b)). Moreover, as dietary DCP levels increased, the transcription of *4e-bp1* was significantly lower than that in the control group (*P* < 0.05) (Figure 7(c)).

## 4. Discussion

Growth performance is one of the crucial indicators to assess effects of FM protein replaced with plant protein. The present results revealed that growth performance of large yellow croaker was significantly affected by dietary DCP levels. WGR and SGR displayed a significant higher value in the DCP20 group (263.91% and 1.85% d^−1^) than the control group (194.79% and 1.54% d^−1^). Meanwhile, WGR and SGR in the DCP40 and DCP60 group were not significantly affected by dietary DCP replacement levels. However, with increasing dietary DCP levels, growth performance was significantly impaired. Dietary DCP replacement levels did not significantly affect SR in the current study. In the present study, according to the broken line regression model analysis of WGR and SGR against dietary DCP replacement levels, the optimal replacement level was recommended to be 8.12% and 9.37% for large yellow croaker, respectively. Results about growth performance also revealed that dietary FM protein could be replaced with less than 60% DCP without significantly inhibiting growth performance under experimental diets and conditions of the present study, which may be due to the fact that as dietary DCP replacement levels increased, the amount of free gossypol in diets was also increased, thus causing damage to the growth performance of fish. These results were similar with relevant research conclusions. Xie et al. [10] found that replacement of FM protein with less than 40% low-gossypol cottonseed protein concentrate could not affect growth performance and SR of swimming crab compared with the FM control group. Zhao et al. [8] indicated that growth performance and SR of rainbow trout were not significantly impacted by the level of 50% concentrated dephenolization cottonseed protein replacing FM protein. However, other studies revealed that growth performance and SR in southern flounder (*Paralichthys lethostigma*) were not significantly impaired when fish fed the diet with 100% low-gossypol cottonseed meal compared with the control diet [6]. Comparable results were investigated in black sea bass (*Centropristis striata*) [7]. These differences in replacement of FM protein with DCP may be due to differences in protein metabolism among aquatic animals fed diets with DCP.

The physical status of aquatic animals related to response to stress, water pollution, and nutritional condition can be evaluated by the analysis of blood parameters [26]. The evaluation of blood biochemistry may also be helpful to the diagnosis of diseases [27]. The current study displayed that as dietary DCP replacement levels increased, the content of TG and TC in fish was not significantly impacted. The trial in rainbow trout was found to have similar results [8]. Fynn-Aikins et al. [28] had well reported that ALT and AST are the most crucial aminotransferases in serum; high activities of ALT and AST indicate hepatic dysfunctions caused by oxidative stress [29]. Results of the present study displayed that the activities of ALT and AST were significant elevated in the DCP20 group compared with the control group and then decreased with the increasing substitution levels of DCP, which is parallel to results of previous study. Yuan et al. [30] indicated that hepatic ALT and AST contents in blunt snout bream significantly increased with 3% dietary cottonseed meal protein hydrolysate (CPH) compared with the FM control group and then significantly decreased as further increasing dietary CPH levels. The elevation of hepatic ALT and AST will promote its transport to the blood.

The increased activities of serum ALT and AST are in accord with the impairment of capacity of hepatic antioxidant. The redox imbalance in body is partly attributed to the excessive generation of reactive oxygen species (ROS), which make metabolism and genetic materials injure [31]. Therefore, SOD and CAT, which are regarded as antioxidant enzymes in fish, are used to scavenge the excessive-production of ROS [32]. Dietary DCP replacement levels significantly elevated the activity of SOD in the DCP20 group compared with the control group. To further investigate the profitable effect on antioxidant capacity of FM protein replaced with DCP, the content of MDA was tested. The peroxidation of lipid will produce MDA, which is also a terrific indicator of oxidative stress in fish [33]. Results displayed that the content of MDA was significantly degraded in the DCP20, DCP40, and DCP80 groups compared with the control group, which is parallel to the result of SOD. Parallel results have been proposed in rainbow trout, which found that dietary FM protein replaced with over 40% concentrated dephenolization cottonseed protein (CDCP) could significantly decrease the content of MDA [8].

The intestine is greatly affected the digestion and absorption of nutrient, and its digestive enzyme activities play an essential role in feed utilization and hence in fish growth [34]. The ability of fish to digest protein in diets is well reflected by the activities of protease in digestive tract [35]. Fish fed diets with from 20% to 80% DCP significantly elevated the activity of trypsin compared with the control group, which revealed that replacement of FM protein with DCP could increase the activity of intestinal trypsin and further promote the absorption and utilization of protein. Trypsin is a selective amino hydrolase, the elevated activity of intestinal trypsin with DCP could be partly attributed to the change of amino acid compositions in diets. The specific mechanism needed to be further explored.

Immunity has a vital impact on resisting external pathogens and maintaining internal homeostasis of animal body, which is made up of nonspecific and specific immunity [36]. Nonspecific immunity is regarded more essential than specific immune in fish due to the imperfect immune response mechanism [37]. Results of the current study displayed that the transcription of proinflammatory cytokine genes (*il-6*, *tnf-α*, and *ifn-γ*) was significantly upregulated in fish fed diets with 20% and 40% DCP. Parallel results were stated in previous research that DCP replacement could induce humoral immune response in fish [38]. These results suggested that the proper substitution level of DCP could activate immune response in large yellow croaker.

It is demonstrated that the TOR pathway makes full use of promoting protein synthesis and regulating growth responding to nutrient availability in eukaryotic organisms [39]. Meanwhile, consumed nutrients such as amino acids also affect regulation of TOR activity in mammals [40]. The promotion of growth performance was contributed to activation of the TOR pathway, which had been observed in some trials of fishmeal protein replaced with cottonseed meal protein at appropriate level [10, 30, 38]. In mammals, ribosomal S6 protein kinase (S6K) and eukaryotic translation initiation factor 4E binding protein (4E-BP1) are widely focused and studied as two the most important TOR substrates [11]. When S6K is phosphorylated, the activity of protein is activated. On the contrary, when 4E-BP1 is phosphorylated, the activity of protein is inactivated. The activation of TOR will phosphorylate S6K and 4E-BP1 so that translation could be activated [41, 42]. Some studies on fish had reported the similar pattern such as largemouth bass (*Micropterus salmoides*) [43] and jian carp [44]. In the current study, the TOR pathway was activated by replacement of FM protein with DCP, and the expression of *tor* was significantly elevated. Identically, dietary replacement of FM protein with DCP significantly elevated the transcription of *s6*, which is the substrate of S6K, but significantly decreased the transcription of *4e-bp1*. These results indicated that dietary FM protein replaced with DCP could activate the transcription of the TOR pathway-related genes. Meanwhile, the improved growth performance in the DCP20 group could be partly attributed to activation of the TOR pathway.

## 5. Conclusion

In brief, the current study first assesses the effects of replacement of FM protein with DCP in large yellow croaker. Based on the broken line regression model analysis of WGR and SGR against dietary DCP replacement levels, the optimal replacement level was recommended to be 8.12% and 9.37% for large yellow croaker, respectively. Results indicated that large yellow croaker fed diets with 20% DCP could improve digestive enzyme activities and antioxidant capacity and further activate immune response and the TOR pathway, thus enhancing growth performance of large yellow croaker.

## Figures and Tables

**Table 1:** Formulation and approximate composition of the experimental diets.

	Diets (g/kg dry matter)				
Ingredients	FM	DCP20	DCP40	DCP60	DCP80
White fish meal^1^	384.00	307.00	230.00	153.00	77.00
Degossypolized cottonseed protein^2^	0.00	89.00	175.00	261.00	349.00
Krill meal^1^	35.00	35.00	35.00	35.00	35.00
Casein	101.00	101.00	101.00	101.00	101.00
Bread flour^1^	256.00	256.00	256.00	256.00	256.00
Soy lecithin	20.00	20.00	20.00	20.00	20.00
Fish oil	51.00	58.00	66.00	73.00	80.00
Choline chloride	2.00	2.00	2.00	2.00	2.00
*α*-Starch	111.00	92.00	75.00	59.00	40.00
Vitamin C	1.00	1.00	1.00	1.00	1.00
Vitamin premix^1^	2.00	2.00	2.00	2.00	2.00
Mineral premix^1^	10.00	10.00	10.00	10.00	10.00
Ca(H_2_PO_4_)_2_	20.00	20.00	20.00	20.00	20.00
Attractant^1^	5.00	5.00	5.00	5.00	5.00
Mould inhibitor^3^	1.00	1.00	1.00	1.00	1.00
Ethoxyquinoline	1.00	1.00	1.00	1.00	1.00
Total	1000.00	1000.00	1000.00	1000.00	1000.00
Proximate analysis (g/kg dry matter)					
Crude protein	418.20	418.90	418.80	418.60	419.70
Crude lipid	120.30	120.10	120.80	120.60	120.40
Crude ash	131.63	130.50	132.75	135.63	131.42
Carbohydrate	165.69	165.69	165.69	165.69	165.69
Gross energy^4^ (kJ/g)	17.49	17.49	17.52	17.52	17.52

^1^The crude protein and crude lipid content and detailed compositions of ingredients were presented in the previous study of He et al. [45]. ^2^Degossypolized cottonseed protein (649.00 g/kg crude protein, 5.00 g/kg crude lipid, 0.06 g/kg free gossypol, 25.40 g/kg valine, 7.40 g/kg methionine, 18.30 g/kg isoleucine, 34.30 g/kg leucine, 18.70 g/kg threonine, 33.10 g/kg phenylalanine, 16.80 g/kg histidine, 24.70 g/kg lysine, and 72.2 g/kg arginine). The degossypolized cottonseed protein was obtained from Da Bei Nong Bio-Tech Co., Ltd., China. ^3^The mould inhibitor is calcium propionate. ^4^The value of gross energy was calculated based on the contents of crude protein, crude lipid, and carbohydrate in diets.

**Table 2:** Primer sequences used for RT-qPCR in the present study^1^.

Target genes	Forward primers (5′-3′)	Reverse primers (5′-3′)	Reference
*il-1β*	CATAGGGATGGGGACAACGA	AGGGGACGGACACAAGGGTA	[21]
*il-6*	CGACACACCCACTATTTACAAC	TCCCATTTTCTGAACTGCCTCT	[21]
*il-8*	AATCTTCGTCGCCTCCATTGT	GAGGGATGATCTCCACCTTCG	[21]
*il-10*	AGTCGGTTACTTTCTGTGGTG	TGTATGACGCAATATGGTCTG	[21]
*tnf-α*	ACACCTCTCAGCCACAGGAT	CCGTGTCCCACTCCATAGTT	[21]
*ifn-γ*	TCAGACCTCCGCACCATCA	GCAACCATTGTAACGCCACTTA	[21]
*tor*	GCTGCAGTGTTGGTGTTGAG	GGACCCTGTCGTCTCGATTC	XM_027288345.1
*s6*	AGAAGCGTATGGCCACTGAG	CAGGAGTGTCCCTTGCTGAG	XM_019267468.2
*4e-bp1*	TGACCATCAACGACTCGGC	CCTGGAATGTTGGGCAGACC	XM_010732553.3
*β-Actin*	GACCTGACAGACTACCTCATG	AGTTGAAGGTGGTCTCGTGGA	[21]

^1^ *il-1β*: interleukin-1*β*; *il-6*: interleukin-6; *il-8*: interleukin-8; *il-10*: interleukin-10; *tnf-α*: tumor necrosis factor-*α*; *ifn-γ*: interferon-*γ*; *tor*: target of rapamycin; *s6*: ribosomal protein S6; *4e-bp1*: eukaryotic initiation factor 4E binding protein 1.

**Table 3:** Effect of degossypolized cottonseed protein on body composition of whole body in juvenile fed the trial diets (g/kg dry matter, means ± SEM, *n* = 3)^1^.

	Diets				
Indexes	FM	DCP20	DCP40	DCP60	DCP80
Moisture (g/kg)	792.00 ± 11.78	814.23 ± 11.99	803.73 ± 19.02	821.17 ± 19.45	810.83 ± 1.53
Crude protein (g/kg)	596.48 ± 7.32	528.37 ± 6.46	584.26 ± 5.61	497.40 ± 9.47	604.60 ± 5.74
Crude lipid (g/kg)	245.35 ± 8.54	245.05 ± 3.11	265.16 ± 10.37	217.40 ± 5.46	228.32 ± 1.68

^1^No repetition of letters in the equal index means significant differences (*P* < 0.05).

**Table 4:** Effect of degossypolized cottonseed protein on serum biochemical indexes of juvenile fed the trial diets (means ± SEM, *n* = 3)^1^.

	Diets				
Indexes	FM	DCP20	DCP40	DCP60	DCP80
TG (mmol/L)	1.61 ± 0.59	2.77 ± 1.03	1.93 ± 0.27	3.19 ± 0.29	1.16 ± 0.39
TC (mmol/L)	1.68 ± 0.28	2.55 ± 0.29	1.77 ± 0.18	2.39 ± 0.17	2.00 ± 0.14
ALT (U/L)	16.82 ± 1.61*b*	35.80 ± 4.17*a*	29.49 ± 3.42*a*	28.35 ± 1.06*a*	25.92 ± 1.47*ab*
AST (U/L)	19.98 ± 7.39*b*	79.27 ± 10.70*a*	49.98 ± 15.14*ab*	31.80 ± 4.84*b*	34.94 ± 3.47*b*
ALP (U/L)	46.47 ± 3.56	42.30 ± 5.63	31.89 ± 10.16	35.74 ± 4.52	39.41 ± 5.26

TG: triglyceride; TC: total cholesterol; ALT: alanine transaminase; AST: aspartate transaminase; ALP: alkaline phosphatase. ^1^No repetition of letters in the equal index means significant differences (*P* < 0.05).

## Data Availability

The data that support the findings of this study are available from the corresponding author upon reasonable request.
